# Depression, antidepressants and driving safety

**DOI:** 10.1186/s40621-017-0107-x

**Published:** 2017-04-03

**Authors:** Linda L. Hill, Vanessa L. Lauzon, Elise L. Winbrock, Guohua Li, Stanford Chihuri, Kelly C. Lee

**Affiliations:** 1 Department of Family Medicine and Public Health, San Diego, USA; 2 Department of Psychiatry, San Diego, USA; 3 Skaggs School of Pharmacy and Pharmaceutical Sciences, University of California, 9500 Gilman Drive, La Jolla, San Diego, CA 92093 USA; 4 Department of Epidemiology, Mailman School of Public Health, New York, USA; 5 Center for Injury Epidemiology and Prevention, Columbia University Medical Center, Columbia University, 722 West 168th Street, New York, NY 10032 USA

**Keywords:** Crashes, Driving, Depression, Antidepressants, Intentional crashes

## Abstract

**Background:**

The purpose of this study was to review the reported associations of depression and antidepressants with motor vehicle crashes.

**Purpose:**

A literature search for material published in the English language between January, 1995, and October, 2015, in bibliographic databases was combined with a search for other relevant material referenced in the retrieved articles.

**Methods:**

Retrieved articles were systematically reviewed for inclusion criteria: 19 epidemiological studies (17 case-control and 2 cohort studies) fulfilled the inclusion criteria by estimating the crash risk associated with depression and/or psychotropic medications in naturalistic settings.

**Results:**

The estimates of the odds ratio (OR) of crash involvement associated with depression ranged from 1.78 to 3.99. All classes of antidepressants were reported to have side effects with the potential to affect driving safety. The majority of studies of antidepressant effects on driving reported an elevated crash risk, and ORs ranged from 1.19 to 2.03 for all crashes, and 3.19 for fatal crashes. In meta-analysis, depression was associated with approximately 2-fold increased crash risk (summary OR = 1.90; 95% CI, 1.06 to 3.39), and antidepressants were associated with approximately 40% increased crash risk (summary OR = 1.40; 95%CI, 1.18 to 1.66).

**Conclusion:**

Based on the findings of the studies reviewed, depression, antidepressants or the combination of depression and antidepressants may pose a potential hazard to driving safety. More research is needed to understand the individual contributions of depression and the medications used to treat depression.

## Review

### Background

Motor vehicles crashes are a leading cause of death in the U.S. with 32,675 fatalities reported in 2014, and an 8% increase in the first half of 2015 (National Highway Traffic Safety Administration. Data: NHTSA [Internet] 2015). Both medical conditions and medications have the potential to impair the ability to safety operate a motor vehicle (Meuleners et al. 2006; Zuin et al. 2002). The crash risk has been reported to increase with increasing numbers of daily medications (LeRoy & Morse 2008; Monarrez-Espino et al. 2014). Depression is a highly prevalent and underdiagnosed (Centers for Disease Control and Prevention 2010; Mitchell et al. 2009) condition. It affects nearly 10% in the adult population, and has a lifetime prevalence of approximately 13% (Hasin et al. 2005; Patten et al. 2006). Psychomotor retardation can be a manifestation of depression and may potentially influence driving safety (Sherwood 1995; Austin et al. 2001; Bulmash et al. 2006). Antidepressants as a group are the second most commonly prescribed drug classes (Aitken 2014). Antidepressants may alleviate the symptoms of depression, but they may be associated with side effects that could impair driving (Sherwood 1995).

The purpose of this study was to review the reported effects of depression and antidepressants on motor vehicle crash risk. The diagnosis and treatment of depression has changed significantly over the last 40 years. This paper includes epidemiologic studies conducted in the last 20 years since the publication of the Diagnostic and Statistical Manual of Mental Disorders, 4^th^ Edition (DSM-IV) in 1994. Experimental studies, mainly using driving simulators, were not included in this review.

## Methods

### Eligibility

Studies were included if they (National Highway Traffic Safety Administration. Data: NHTSA [Internet] 2015) examined the association between depression or antidepressant use and motor vehicle crash risk; (Meuleners et al. 2006) used an epidemiologic design (e.g., cohort, case control, and case-crossover), and (Zuin et al. 2002) were published in English between January, 1995, and October, 2015. Cross-sectional studies, qualitative studies, reviews, commentaries, opinion pieces, and magazine articles were excluded. In this review, depression was ascertained through questionnaires such as the Geriatric Depression Scale (GDP), General Health Questionnaire (GHQ) or their modified versions, as well as interviews and claims-based databases. Antidepressants included classes of medications prescribed for the treatment of depression such as tricyclics (e.g. amitriptyline, imipramine), monoamine oxidase inhibitors (MAOI) (, e.g. phenelzine, tranylcypromine), selective serotonin reuptake inhibitors (SSRI) (, e.g.citalopram, escitalopram), serotonin-norepinephrine reuptake inhibitors (SNRI )(e.g.venlafaxine, duloxetine), and atypicals (e.g. bupropion, mirtazapine).

### Search Strategy and Data Extraction

Relevant articles were identified through a comprehensive search of the research literature. Electronic database searches included PubMed (1995-October, 2015), Medline Plus (1995-October, 2015), Google Scholar (1995-October, 2015), Ingenta Connect (1995-October, 2015), and grey literature databases. One author (LH) conducted the initial search and screened study titles and abstracts using the inclusion and exclusion criteria. Other authors (SC, KL, EW) independently searched for relevant studies and collected and recorded data used to estimate odds ratios from eligible studies. For each included study, information on author, year of publication, study sample, study design, country, study period, and findings were noted.

### Quality Assessment and Data Analysis

**Fig. 1:**
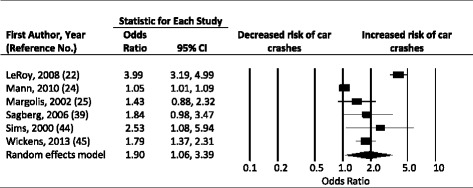
Forest plot, summary odd ratio and 95% confidence of association of risk of car crashes with depression. The summary odds ratio is indicated by the diamond. *Horizontal bars* indicate the 95% confidence intervals

**Fig. 2:**
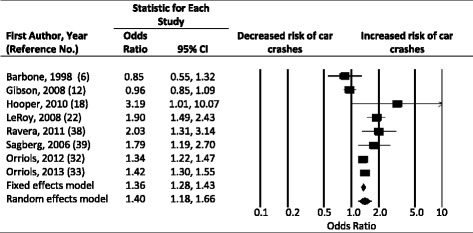
Forest plot, summary odd ratio and 95% confidence of association of risk of car crashes with anti-depressants. The summary odds ratio is indicated by the diamond. *Horizontal bars* indicate the 95% confidence intervalsᅟ

All studies that met the inclusion criteria were assessed for quality using the Newcastle-Ottawa Scale (NOS) (Wells et al. 2015) as recommended by the Cochrane Collaboration on bias assessment (Higgins & Green S. Cochrane Handbook for Systematic Reviews of Interventions Version 5.1.0 [updated March 2011). For the study designs included in this study, the best possible score is 9. Better quality studies will have higher scores. Heterogeneity was assessed using the Q and I^2^ tests, with P ≤ .05 and I^2^ > 0.5 considered homogenous. Funnel plots were used to assess publication bias. Data abstracted from each study were entered in the Comprehensive Meta-Analysis software (Borenstein et al. 2005) to compute the individual odds ratios and a summary odds ratio for each of the two analyses. A fixed effects model was used unless heterogeneity was present, in which case, a random-effects model would be preferred. Two forest plots were created; one to show the distribution of the effect of depression on car crash risk (Fig. 1) and another one to show the distribution of the effect of antidepressant use on car crash risk (Fig. 2).

## Results

### The effect of depression on driving and crashes

Seven studies investigating the effect of depression on driving met the inclusion criteria (Table 1). Rainio et al (Rainio et al. 2007) conducted a retrospective review of crashes in 2001 and 2002, using interviews of police, surviving drivers, and family members, medical records, and autopsy reports. A physician on the team made the determination of the presence of depression. This Finnish study used the reports of the road crash investigation teams which investigate every fatal crash in Finland. Of the 640 crash deaths analyzed, 390 were drivers considered to be at fault. Of these 390, 6.4% had depression, while one non-fault driver and none of the non-fault passengers had depression (Rainio et al. 2007). No medication data were included in the analysis. Sagberg (Sagberg 2006) used self-report questionnaires from 4448 crash-involved drivers in Norway and found an OR of 2.43 in persons reporting depression. Though antidepressants were included, the relationship between antidepressants and depression was not included in the analysis. Mann et al (Mann et al. 2010) did a cross-sectional telephone survey of adults in Ontario, Canada aged 18 and older. Depression and anxiety were determined using two subscales of the 12-item General Health Questionnaire: Depression-anxiety and social functioning. With a sample of 4935 adults, Mann et al (Mann et al. 2010) found the odds of crash involvement increased significantly with an increase in the anxiety/depression score, with a five percent increase in the risk of crash involvement for every unit of anxiety/depression increase (95% CI, 1.01 to 1.09). Medications for depression were not included in the study. Wickens et al (Wickens et al. 2013), also with an Ontario sample of 12,830, studied self-reported anxiety and mood disorders, queried via telephone survey, and found an increase in crashes (OR = 1.78; 95% CI, 1.37 to 2.31). There was no report of antidepressant use. Sims et al (Sims et al. 2000), in a prospective analysis of 174 older adults, used baseline and one year follow-up in-person assessments. They found that a Geriatric Depression Scale score ≥16 was associated with an increased crash rate (RR =2.53; 95% CI,1.08 to 5.95). While antidepressant use was assessed, the relationship between depression, antidepressants and crashes was not reported, and in this study the relationship between antidepressant use and crashes was not significant. LeRoy (LeRoy & Morse 2008) found an OR of 3.99 for crash risk with depression in a case control study of 81,408 cases and 244,224 controls (95% CI, 3.19 to 4.99) using claims-based population data. Another study of older adults (Margolis (Margolis et al. 2002)), using the Geriatric Depression Scale, included depression in their analysis and found no association; however, this study was small, with a low prevalence of depression in the study population (Margolis et al. 2002; McGwin et al. 2000).

**Table 1:** Depression and overall crash rate

Lead Author	Year of Publication	Country	Sample Size	Methodology (data collection period)	Confounders	Findings
(Rainio et al. 2007)	2007	Finland	542 crashes, 640 crash deaths	Retrospective review of crashes, autopsy records (2001, 2002)	Adjusted: Age, survival from accident, driver/passenger status Non-Adjusted: Retrospective review of medical records; surviving non-A parties and passengers excluded as controls; no medication data were included in analyses; incidence of undiagnosed depression or other psychiatric disorders is unknown	Of fatal crashes, 6.4% of killed A-parties, (key driver), 3.9% of surviving A-parties (key survivor) and 1.5% of killed non-A-parties (non-key driver) had depression; 0% of killed passengers had depression
(Sagberg 2006)	2006	Norway	4448 crash involved drivers	Self-report questionnaire of drivers in the last 6 months (no year reported)	Adjusted: Gender, age, driving distance, other involved road user, crash type, responsible for multiple vehicle crashes Non-Adjusted: Low response rate; biased or under-reporting from self-reports; self-report of depressive symptoms not confirmed; exclusion of diseases such as epilepsy	“Feeling blue, depressed” on self-report had OR of 2.43 of being at fault, p = .03
(Mann et al. 2010)	2010	Canada	4935 adult drivers	Cross-sectional phone survey (2002-2004)	Adjusted: Alcohol and cannabis lifetime and recent use; driving exposure and stressful driving environment Non-Adjusted: Driving exposure and collision involvement measured by self-report; no report of medication use for depression; comorbid psychiatric or medical conditions not captured	Risk of collision involvement increased with each unit of depression-anxiety score using General Health Questionnaire (GHQ-12) (OR = 1.05, CI: 101-1.09)
(Wickens et al. 2013)	2013	Canada	12,830	Telephone survey; motor vehicle accidents in past 12 months (conducted between 2002-2009)	Adjusted: Age, gender, driving exposure, driving after alcohol or cannabis use Non-Adjusted: Biased or under-reporting from self-reports; no report of medication use for depression and anxiety disorders; driving after use of substances other than alcohol or cannabis not studied	Self-report of collision involvement of those with probable mood and anxiety disorder had OR = 1.78 (CI: 1.37, 2.31)
(Margolis et al. 2002)	2002	US	1416 women 65-84	Prospective analysis of older drivers (1991-1996)	Adjusted: Driving miles per week, number of motor vehicle crashes; use of medications and alcohol; functional status, visual acuity; previous history of falls Non-Adjusted: Sample limited to white women aged 65 years and older and living in Portland, OR; comorbid psychiatric conditions not captured; cases limited to motor vehicle crashes that resulted in a police report	Depression did not predict crash (only 3.4% of participants had depression per Geriatric Depression Scale)
(Sims et al. 2000)	2000	US	174 older adults; 61 subjects had crashes during study period	Prospective analysis of incident crash (1991-1992)	Adjusted: Age, race, gender, days per week driven Non-Adjusted: Small sample size; biased or under-reporting from self-reports of health status, medical diagnoses and medications; adherence to medications unknown; mileage driven were extrapolated based on average annual driving miles reported by subjects	6.9% of sample had Geriatric Depression Scale ≥16; which increased risk of crash (RR = 2.53,CI:1.08-5.95, *p* = 0.03)
(LeRoy & Morse 2008)	2008	US	81,408 cases and 244,224 age-, sex-and date-matched controls	Case-Control (1998-2002)	Adjusted: Age, gender, 6 months of coverage. Non adjusted: medical diagnosis and medication relationship.	OR = 3.99 (CI:3.19, 4.99), *p* < .001

*OR* odds ratio, *CI* confidence interval, *RR* relative risk

### The effect of antidepressants on driving

Ten studies (Table 2) meeting inclusion criteria evaluated the relationship between antidepressants and motor vehicle crashes. Several studies have used population-based data sets to assess the risk of antidepressants and driving (National Highway Traffic Safety Administration. Data: NHTSA [Internet] 2015; Meuleners et al. 2006; Aitken 2014; Bramness et al. 2008; Barbone et al. 1998; Ravera et al. 2011; Gibson et al. 2009; Lam et al. 2005; Greenblatt et al. 1999). Hooper et al (Hooper et al. 2010) retrospectively studied a U.S. active duty military population between 2002-2006, using an integrated health systems database, and found an adjusted OR of 3.19 for the effect of antidepressants on crash risk (95% CI, 1.01 to 10.07). Depression was not assessed. Orriols et al (Orriols et al. 2012) did a case-crossover analysis of 72,685 drivers in France using the national health care insurance database and the national police database and found an OR of 1.34 for crash and prescription of antidepressants as a class (95% CI, 1.22 to 1.47). SSRIs (OR = 1.30; 95% CI, 1.16 to 1.46), SNRIs (OR = 1.51; 95% CI, 1.25 to 1.84), and other antidepressants (OR = 1.30; 95% CI, 1.01 to 1.67) also had a significant association with increased risk of crash. In a different analysis, Orriols et al (Orriols et al. 2013) studied 109,406 older drivers aged 66-84 years in Quebec, Canada, using provincial police and health databases. They found that 20% had at least one prescription of an antidepressant during the study period, and 2.7% were exposed to antidepressants on the day of the crash (OR = 1.19; 95% CI, 0.94 to 1.51). Neither of the studies by Orriols included data on depression. Rapaport (Rapoport et al. 2011) did a case-only time to event analysis in 159,678 persons in Ontario, also using provincial databases, and found that SSRI and SNRI antidepressants were associated with at-fault crashes alone (HR = 1.10; 95% CI, 1.09 to 1.13), in combination with a benzodiazepine (adjusted HR = 1.23; 95% CI, 1.17 to 1.28), and with an anticholinergic medication (adjusted HR = 1.63; 95% CI, 1.57 to 1.69). The period within the first 3-4 months of antidepressant initiation also was associated with increased risk of crash (Rapoport et al. 2011). Bramness (Bramness et al. 2008), using national databases, found non-sedating antidepressants increased the risk for traffic crashes (SIR = 1.6; 95% CI, 1.5 to 1.7) more than sedating antidepressants (SIR = 1.4; 95% CI, 1.2 to 1.6). Neither Rapaport nor Bramness include depression as a variable. Sagberg (Sagberg 2006), in a Norwegian study of 4,448 crash involved drivers, found the self-reported use of antidepressants had an adjusted OR for crash of 1.70 (95% CI, 0.98 to 2.24). While this study, and the following two studies (LeRoy and Sims) included both depression and anti-depressants, the relationship between the two variables was not assessed. LeRoy (LeRoy & Morse 2008), in a US-based case-control study of 5,398 cases and 16, 194 controls, using national survey and also claims-based databases, found that serotonin-2 antagonist/reuptake inhibitors had an OR for crash of 1.90 (95% CI, 1.49 to 2.44). Barbone (Barbone et al. 1998), in a case crossover study in the UK, used a local prescription database and found no association between crash and tricyclic or SSRI antidepressants in the 1998 study of 19,386 crashes. Ravera (Ravera et al. 2011) in the Netherlands study of 3,963 cases and 18,828 controls used three existing Dutch population-based databases and found an OR of crash with SSRI of 2.03 (95% CI, 1.31 to 3.14). Gibson (Gibson et al. 2009), using a primary care database, found that SSRI had no effect in the short term (IRR = .92; 99% CI, 0.75 to 1.12), but had a small increase in the risk of crash with extended use (IRR = 1.16; 99% CI, 1.06 to 1.28) in a case crossover study of 49,821 crashes. None of these three studies included depression as a variable.

**Table 2:** Antidepressants and crash risk^a^

Lead author	Year of publication	Country	Sample size	Methodology	Confounders	Findings
(Orriols et al. 2012)	2012	France	34,896 cases, 37,789 controls	Case-control to compare drivers responsible for crash and those not responsible for crash and case crossover analysis to compare exposure immediately before crash with exposure during earlier period (2005-2008)	Adjusted: Gender, age, socioeconomic category, concomitant use of high risk medications, injury severity, blood alcohol concentration, use of sedative-hypnotics, time of day, accident type, responsibility of driver Non-Adjusted: Data limited to those filed in police reports or database; antidepressant drug adherence not confirmed; lack of information on medical/psychiatric diagnoses	4% of all drivers exposed to 1 antidepressant on day of crash. Antidepressant and risk of being responsible for crash: All antidepressants (OR = 1.34, CI:1.22, 1.47), TCAs (OR = 1.05, CI:0.81-1.36), SSRI (OR = 1.30, CI: 1.16-1.46)*, SNRI (OR = 1.51, CI: 1.25-1.84) *p* < .0001, other antidepressants (OR = 1.30, CI:1.01-1.67) (p < .05) During period immediately before rash, increased risk of crash higher among antidepressant users with only 1 prescription (OR = 1.45, CI: 1.24-1.79)* and changes in antidepressant treatment (OR = 1.32, CI: 1.09-1.60)
(Orriols et al. 2013)	2013	Canada	109,406 (aged 66-84)	Case cross-over analysis of those exposed to antidepressants immediately before road traffic crash and those exposed during earlier periods (first road crash between 1988-2000)	Adjusted: Duration of treatment with antidepressant Non-Adjusted: Antidepressant drug adherence not confirmed; no confirmation of psychiatric diagnoses corresponding to antidepressant prescription; no information on use of alcohol or illicit substances prior to crash in either group	2.7% exposed to antidepressant on day of crash and 20.1% had at least one antidepressant prescription over study period. Antidepressant prescription before crash increased risk of crash (OR = 1.19, CI: 1.08, 1.30) compared to antidepressant exposure 4-8 months before crash.
(Rapoport et al. 2011)	2011	Canada	159,678 (age ≥65)	Population based case-only time to event (motor vehicle crash after 66^th^ birthday) analysis (2000-2007)	Adjusted: Gender, number of license suspensions before first collision, medication burden Non-Adjusted: Limited to subjects 65 and older; antidepressant drug adherence not confirmed; no information on use of alcohol or illicit substances prior to crash	5% exposed to antidepressant in month prior to crash. Second generation antidepressants increased risk of crash (HR = 1.10,CI: 1.07, 1.13, *p* < 0.0001); benzodiazepines increased risk (adjusted HR = 1.05 (CI: 1.03-1.07, *p* < 0.0001) similarly to antidepressants. Increased risk apparent for first 3-4 months after antidepressant started.
(Sagberg 2006)	2006	Norway	4448 crash-involved drivers	Case-control study using self-reported questionnaires	Adjusted: Crash type, responsible for crash, psychiatric/medical illnesses, medication classes, symptoms that may influence car crash, driving experience Non-Adjusted: Self-reports could underestimate use of antidepressants or presence of symptoms; medication adherence not confirmed; no confirmation of psychiatric/medical diagnoses; no information on use of alcohol or illicit substances	Use of antidepressants also increased risk (OR = 1.70, CI: 0.98-2.24)
(Bramness et al. 2008)	2008	Norway	Of road accidents (20,494) with personal injuries, 204 drivers exposed to sedating antidepressants, 884 drivers exposed to non-sedating antidepressants (18-69 years)	Retrospective analysis (2004- 2006)	Adjusted: Gender, age, Non-Adjusted: Antidepressant drug adherence not confirmed; lack of information on medical/psychiatric diagnoses; no information on alcohol or substance use; no information on driver responsibility or accident severity	Sedating antidepressants increased risk for traffic accidents (SIR = 1.4, CI:1.2, 1.6) and non-sedating antidepressants increased risk for traffic accidents (SIR = 1.6, CI:1.5, 1.7). SIR did not change for different time periods after prescription dispensing, concomitant medication use or for new users.
(Hooper et al. 2010)	2010	US	962 fatal motor vehicle crash (cases) and 2886 (controls) (active duty military population)	Case-Control (2002-2006)	Adjusted: Gender, age, branch of service, rank, deployment Non-Adjusted: Subjects limited to active duty military personnel; medication adherence not confirmed; medication consumed outside military health system not captured	Antidepressants were an independent mediator of fatal motor vehicle crashes (adjusted OR = 3.19,CI: 1.01, 10.07); “other mental disorders” also increased risk (adjusted OR = 2.28, CI: 1.41, 3.70)
(LeRoy & Morse 2008)	2008	US	5398 cases, 16,194 controls >50	Case-Control (1998-2000 and 1998-2002)	Medication adherence not confirmed; duration of treatment not captured	Serotonin-2 antagonist/reuptake inhibitors (OR = 1.90,CI: 1.49, 2.44) SNRI: (OR = 1.78,CI: 1.19, 2.66)
(Gibson et al. 2009)	2009	UK	49821	Case Cross-over study	Adjusted: Age, time of exposure to medication classes Non-Adjusted: Medication adherence not confirmed; lack of information on medical/psychiatric diagnoses; use of alcohol and other substances not captured	SSRI short term use IRR = .92 (99% CI: .75-1.12); extended use IRR = 1.16 (99% CI: 1.06-1.28).
(Barbone et al. 1998)	1998	UK	19386 crashes	Case Cross-over study	Adjusted: Age, gender, severity of injuries, time of day, lighting conditions, number of vehicles involved, driver at fault, breath alcohol test Non-Adjusted: Medication adherence not confirmed; lack of information on medical/psychiatric diagnoses	TCA and SSRI antidepressants had no association. TCA OR = .93 (CI:.72-1.21); SSRI OR = .85 (CI: .55-1.33)
(Ravera et al. 2011)	2011	Netherlands	3963 cases 18828 controls	Case-control study	Adjusted: Age, gender, season, weather, time of accident, lighting conditions, severity of accident Non-Adjusted: Medication adherence not confirmed; cases limited to those who required medical assistance from the traffic accident; cases limited to those who were negative for alcohol use; lack of information on medical/psychiatric diagnoses	SSRI OR = 2.03 (CI:1.31-3.14)

^a^ *SIR* standardized incidence ratios, *CI* confidence interval, *OR* odds ratio, *SSRI* selective serotonin reuptake inhibitor, *TCA* tricyclic antidepressants, *SNRI* serotonin norepinephrine reuptake inhibitors

### Meta-analysis: Depression and car crash risk

Three cohort (Mann et. al (Mann et al. 2010); Margolis et. al (Margolis et al. 2002); Sims et.al (Sims et al. 2000)) and three case control studies (LeRoy et. al (LeRoy & Morse 2008); Sagberg (Sagberg 2006); Wickens et. al (Wickens et al. 2013)) that examined the association between depression and car crash risk were included in the quantitative meta-analysis (Table 1). One study (Rainio et. al (Rainio et al. 2007)) was excluded from the depression meta-analysis because it assessed aggregate data on crashes, crash deaths, and depression. Effect estimates showed some heterogeneity (Q = 153.741, df = 5, *P* = 0.000; I^2^ = 96.748), hence a random effects model was used. A funnel plot from the six studies did not indicate any major publication bias. Pooled data from the six studies indicate that depression nearly doubles the risk of involvement in a car crash (summary OR = 1.90; 95% CI,1.06 to 3.39) (Fig. 1).

### Meta-analysis: Antidepressant use and car crash risk

Three case-crossover (Barbone et.al (Barbone et al. 1998), Gibson et. al (Gibson et al. 2009), Orriols et. al (Orriols et al. 2013)) and five case control studies (Hooper et. al (Hooper et al. 2010), LeRoy et. al (LeRoy & Morse 2008), Ravera et.al (Ravera et al. 2011), Sagberg et.al (Sagberg 2006), Orriols et. al (Orriols et al. 2012)) were included in the quantitative meta-analysis on antidepressant use and crash risk (Table 2). Two studies (Bramness et. al (Bramness et al. 2008), Rapoport et. al (Rapoport et al. 2011)) were excluded from the antidepressant use meta-analysis because event data could not be extracted. Effect estimates showed some heterogeneity (Q = 47.337, df = 7, P = 0.000; I^2^ = 85.212), hence a random effects model was used. The random effects and the fixed effects model estimates were close. A funnel plot from the six studies did not indicate any major publication bias. Pooled data from the eight studies show that antidepressant use may increase the risk of car crash involvement by 40% (summary OR = 1.40; 95% CI, 1.18 to 1.66) (Fig. 2).

## Discussion

Depression and antidepressants have mental and physical effects with the potential to adversely affect the ability to operate a motor vehicle. Depression, in addition to psychomotor retardation, is often associated with suicidal ideation and intent, increasing the potential for both unintentional and self-harm related motor vehicle crashes (Lam et al. 2005). The studies reviewed, conducted mainly in developed countries, found associations between depression and crashes. Though estimates are hampered by the variation in study population and study design, depression was generally found to approximately double the risk of crash risk.

Antidepressants have numerous side effects that include drowsiness, hypotension, suicidal ideation, dizziness, decreased seizure threshold, nausea, and anxiety. These may individually and combined have the potential to interfere with driving abilities. Studies of the effects of antidepressants as a class and driving found a modest increase in crash risk, with OR ranging from 1.19 to 1.90 for crashes, and 3.19 for fatal crashes. The effect varied by type of antidepressant, with significant variation between studies, but averaged about 1.4 times the crash risk in meta-analysis.

Antidepressants have potentially conflicting contributions to motor vehicle crashes in relieving the effects of depression and suicide while posing side effects that may affect driving. While the benefits of antidepressants outweigh their potential risks, prospective studies are needed to better understand the risk of antidepressants and depression on motor vehicle crashes. Of the seven studies included in this analysis assessing the effects of depression, only three (Sagberg, LeRoy and Sims) included information on antidepressants. However, no comparison of depression scores, medications use, and crash risk in given individuals was reported. Equally, the studies of the effects of antidepressants did not assess the current or past levels of depression. Future studies are needed to control for these interactions.

In addition to the effects of the drugs when taken alone, antidepressants can interact with numerous classes of medications primarily due to their inhibition of metablism of other drugs that are cleared through the cytochrome P-450 system of enzymes. Drug interactions may be especially important in impairing attention and cognition when antidepressants are combined with drugs that also have sedative properties, such as benzodiazepines and tricyclic antidepressants (TCAs). Among antidepressants and their active metabolites, norfluoxetine and fluvoxamine have significant inhibitory effects on CYP 3A4 isoenzyme, which is the most abundant CYP enzyme found in the human body (Greenblatt et al. 1999). Fluoxetine and fluvoxamine have been reported to reduce metabolism of multiple drugs, specifically the triazolobenzodiazepines (triazolam, alprazolam, and midazolam). By blocking the metabolism of these benzodiazepines, the serum concentrations of the benzodiazepines may increase and have increased side effects (i.e. increased sedation, dizziness, impaired cognition). It should be recognized that although *in vitro* affinities of antidepressants for the respective isoenzymes can be very helpful for predicting potentially dangerous drug combinations, there is wide variability between patients and their susceptibility for these interactions. Much of this variability can be attributed to genetic polymorphisms.

The strengths of this review include that 17 of the studies reported used large population-based databases, and 14 included detailed crash analysis, increasing the validity of their findings. Also included were studies from across a number of developed countries, with well-designed studies that met the inclusion criteria. The studies included in this review have several limitations. The criteria for determination of depression ranged from self-report to claims-based diagnostic codes. In determining the effects of depression and antidepressants, it is difficult to distinguish effects of depression from effects of drugs. The distinction is hampered by the retrospective methodology of the majority of the studies. Additionally, antidepressants are often prescribed with other psychotropic medications, increasing the potential for crashes due to both drugs, as has been demonstrated in several studies (Rapoport et al. 2011).

Due to the limitation of these studies, the extent to which antidepressants mitigate the effects of depression remains unknown. However, the larger association of depression with crash risk, vs. the use of antidepressants, suggests that treatment of depression is likely to reduce the risk. In the management of depression the risk-benefit ratio fo treatment should be considered, as well as the side effect profile when medications are being considered.

## Conclusions

Based on the reports of the studies included in this analysis, depression and antidepressants pose a potential hazard to driving safety. Physicians and other health providers, including pharmacists, should recognize the inherent risks of both the disorder and medications on driving and educate their patients accordingly. More research is needed to understand the individual contributions of depression versus the medications used to treat depression and to identify strategies to mitigate the effect of both on driving safety.

## Abbreviations

CI: Confidence interval
DSM-IV: Diagnostic and Statistical Manual of Mental Disorders
GDP: Geriatric Depression Scale
GHQ: General Health Questionnaire
MAOI: Monoamine oxidase inhibitors
OR: Odds ratio
SNRI: Serotonin-norepinephrine reuptake inhibitors
SSRI: Selective serotonin reuptake inhibitor
TCA: Tricyclic antidepressants
